# Tau antibody 77G7 targeting microtubule binding domain suppresses proteopathic tau to seed tau aggregation

**DOI:** 10.1111/cns.13970

**Published:** 2022-09-17

**Authors:** Longfei Li, Jin Miao, Dandan Chu, Nana Jin, Yunn Chyn Tung, Chun‐Ling Dai, Wen Hu, Cheng‐Xin Gong, Khalid Iqbal, Fei Liu

**Affiliations:** ^1^ Department of Neurochemistry Inge Grundke‐Iqbal Research Floor New York State Institute for Basic Research in Developmental Disabilities Staten Island New York USA; ^2^ Key Laboratory of Neuroregeneration of Jiangsu and Ministry of Education Nantong University Nantong China; ^3^ Laboratory Animal Center Nantong University Nantong China

**Keywords:** Alzheimer's disease, immunotherapy, propagation of tau pathology, tau, tau pathology

## Abstract

**Introduction:**

Neurofibrillary tangle (NFT) of hyperphosphorylated tau is a hallmark of Alzheimer's disease (AD) and related tauopathies. Tau lesion starts in the trans‐entorhinal cortex, from where it spreads to limbic regions, followed by neocortical areas. The regional distribution of NFTs associates with the progression of AD. Accumulating evidence suggests that proteopathic tau can seed tau aggregation in a prion‐like fashion in vitro and in vivo. Inhibition of tau seeding activity could provide a potential therapeutic opportunity to block the propagation of tau pathology in AD and related tauopathies.

**Aims:**

In the present study, we investigated the role of 77G7, a monoclonal tau antibody to the microtubule‐binding repeats, in repressing the seeding activity of proteopathic tau.

**Results:**

We found that 77G7 had a higher affinity toward aggregated pathological tau fractions than un‐aggregated tau derived from AD brain. 77G7 inhibited the internalization of tau aggregates by cells, blocked AD O‐tau to capture normal tau, and to seed tau aggregation in vitro and in cultured cells. Tau pathology induced by hippocampal injection of AD O‐tau in 3xTg‐AD mice was suppressed by mixing 77G7 with AD O‐tau. Intravenous administration of 77G7 ameliorated site‐specific hyperphosphorylation of tau induced by AD O‐tau in the hippocampi of Tg/hTau mice.

**Conclusion:**

These findings indicate that 77G7 can effectively suppress the seeding activity of AD O‐tau and thus could be developed as a potential immunotherapeutic drug to inhibit the propagation of tau pathology in AD and related tauopathies.

## Graphical abstract

Tau monoclonal antibody 77G7 targets microtubule binding repeats of tau. 77G7 displayed a higher affinity toward pathological tau. 77G7 inhibited the internalization of tau aggregates by cells, blocked oligomeric tau derived from AD brain to capture normal tau and to seed tau aggregation in vitro, in cultured cells and in vivo. Thus, 77G7 could be developed as a potential immunotherapeutic drug to inhibit the propagation of tau pathology in AD and related tauopathies.

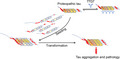

## 1 BACKGROUND

Alzheimer's disease (AD) is one of the most devastating neurological diseases of the brain. Currently about 5.8 million people in the United States alone are victims of AD. This number is projected to rise to nearly 14 million by 2050 if no treatment to prevent or inhibit this disease is found (https://alz.org/alzheimers‐dementia/facts‐figures). AD is characterized by extracellular plaques of amyloid β‐peptide (Aβ) and intraneuronal neurofibrillary tangles (NFTs) of abnormally hyperphosphorylated tau.^1^, ^2^, ^3^ The number of NFTs, but not of Aβ plaque load, is positively correlated with the clinical symptoms of AD,^4^, ^5^, ^6^, ^7^ indicating a fundamental role of tau pathology in AD pathogenesis. In addition, tau lesion is also observed in other neurodegenerative disorders, such as progressive supranuclear palsy (PSP), corticobasal degeneration (CBD), Pick's disease (PiD), and frontotemporal dementia (FTD). These disorders are collectively termed as tauopathies.^8^

By using autopsied human brains, Braak and Braak reported that tau lesion in AD brain starts in the trans‐entorhinal cortex, from where it spreads to limbic regions, followed by neocortical areas.^9^, ^10^ However, recent works have demonstrated that individual tau patterns do not fit neatly into the Braak staging system.^11^ The distribution of tau pathology associates with the progression of this disease.^10^, ^12^ Recently, similar stages were shown by tau tracer retention measured with positron emission tomography.^13^, ^14^, ^15^ Thus, tau pathology in AD brain may spread along neuroanatomical connections, which underlies the progression of AD.^16^

Spread of tau pathology was replicated in vivo in animals. Injection of tau aggregates from Tau_P301S_ mouse brain into wild‐type mouse brain was found to induce tau pathology not only at injection site but also in the synaptic connected region,^17^ which led to a concept of propagation of tau pathology. Following this study, tau aggregates generated in vitro or isolated from brains of AD or other human tauopathies, and tau transgenic mice are reported to template tau aggregation in cultured cells^18^, ^19^ and to induce tau pathology in animal brains,^20^, ^21^, ^22^, ^23^, ^24^, ^25^ suggesting that aggregated tau serves as proteopathic tau seeds and may template tau aggregation in a prion‐like fashion.

Tau pathology correlates directly with the degree of cognitive impairment, which makes tau immunotherapy as a potential treatment approach for AD. It was found that immunotherapy with tau antibodies could effectively target pathogenic tau species, promote their clearance, halt the progression of tau pathology and reverse cognitive impairment in mice.^26^, ^27^, ^28^, ^29^, ^30^ Up to date, over dozen tau immunotherapies have entered clinical trials. Some of these trials have failed, but most of the failed antibodies target the N‐terminus of tau.^31^, ^32^ We developed a monoclonal antibody 77G7 to the microtubule‐binding domains of tau and found it strongly reacted with tau from AD brain.^33^ In the present study, we found that antibody 77G7 displayed higher affinity to pathological tau than to normal tau, blocked the seeding activity of oligomeric tau derived from AD brain (AD O‐tau) and inhibited the binding of normal tau to AD O‐tau. Mixing AD O‐tau with 77G7 can suppress it to induce tau pathology in hippocampus of 3xTg‐AD mice. Furthermore, intravenous administration of 77G7 ameliorated hyperphosphorylation of tau induced by AD O‐tau in Tg/hTau mouse hippocampi.

## 2 MATERIALS AND METHODS

### 2.1 Animals

The 3xTg‐AD mice were purchased from the Jackson Laboratory (New Harbor, ME, USA; https://www.jax.org/strain/004807) and then bred in our Institute. The hemizygous human tau transgenic mice with murine tau knockout background (Tg/hTau, Jackson stock number 005491, B6.Cg‐*Mapt* ^*tm1(EGFP)Klt*^ Tg(MAPT)8cPdav/J)^34^ were obtained from Jackson Laboratory (Bar Harbor, ME, USA). Animals had access to food and water ad libitum. All animal handling and use were as per the protocol approved by our Institutional Animal Care and Use Committee in accordance with the PHS Policy on Human Care and Use of Laboratory Animals.

### 2.2 Monoclonal 77G7 antibody generation

#### 2.2.1 Immunization of animals

Mouse monoclonal tau antibody 77G7 were generated at our institute. Briefly, recombinant human tau 441 purified from Escherichia coli was used as the immunogen. Tau was emulsified in the presence of complete Freund's adjuvant (1:1 vol/vol; Difco Laboratories, Detroit, MI, USA) with the minichanger of an Omni Mixer (Omni International, Kennesaw, GA, USA) at 4°C. BALB/cJ mice (7 weeks old; The Jackson Laboratory, Bar Harbor, ME, USA) were inoculated intradermally and subcutaneously with tau as previously described.^35^ The animals were bled retro‐orbitally and monitored for antibody response by the ELISA procedure.

#### 2.2.2 Production of hybridomas

Spleen cells were fused to the NS0 myeloma cell line. Initial screening of hybridoma supernatants from 96‐well plates was performed by ELISA. Each selected hybridoma was cloned three times by limiting dilution and used to produce ascites in 2,6,10,14‐tetramethylpentadecane (Pristane; Sigma‐Aldrich)‐primed mice. An Ig subtype identification kit (Boehringer Mannheim Biochemical, Indianapolis, IN, USA) was used for isotyping the antibody.

#### 2.2.3 Epitope mapping

Epitope mapping of each tau antibody was carried out using 15‐mer overlapping synthetic tau peptide manufactured by Pepscan (Lelystad, The Netherlands).

### 2.3 Plasmids, antibodies, and other reagents

pCI/hemagglutinin (HA)‐tau_151–391_ and pGEX6P1/tau_151–391_ were constructed as described previously.^36^ The primary antibodies used in the present study are listed in Table 1. Horseradish peroxidase (HRP)‐conjugated anti‐mouse and anti‐rabbit IgGs were obtained from Jackson ImmunoResearch Laboratories (West Grove, PA, USA). Alexa 488 and Alexa 555‐conjugated‐secondary antibody were from Life Technologies (Rockford, IL, USA).

**TABLE 1:** Primary antibodies used in this study

Antibody	Type	Specificity	Species	Source/reference (cat/lot)
43D	Mono‐	Pan‐tau (a.a6‐18)	M	In house/Biolegend (816601)
R134d	Poly‐	Total tau	R	In house^33^
Tau5	Mono‐	tau (a.a210‐230)	M	Millipore (MAB361/1816394)
77G7	Mono‐	tau (a.a.244–368)	M	In house/Biolegend (816701)
Anti‐pS199	Poly‐	p‐tau (S199)	R	Invitrogen (44734G/0300A)
AT8	Mono‐	p‐tau (S202/T205)	M	ThermoScientific (MN1020/PI205175)
Anti‐pT212	Poly‐	p‐tau (T212)	R	Invitrogen (44740G/1709582A)
Anti‐pS214	Poly‐	p‐tau(S214)	R	Invitrogen (44742G/0500B)
Anti‐pT217	Poly‐	p‐tau(T217)	R	Invitrogen (44,744/785771A)
Anti‐pS262	Poly‐	p‐tau(S262)	R	ThermoScientific (44‐750G/0204)
PHF1	Mono‐	p‐tau(S396/404)	M	Dr. Peter Davies
Anti‐pS422	Poly‐	p‐tau(S422)	R	Invitrogen (44764G/2063690)
GAPDH	Poly‐	GAPDH	R	Sigma (G9545/015M4824V)
92e	Poly‐	tau	R	In house
T22	Poly‐	Oligomeric tau	R	Millipore (ABN454/3055586)
β‐tubulin	Poly‐	β‐tubulin‐III	R	Convince (DRB‐435P‐100)
HA	Poly‐	HA	R	Sigma (H6908/115M4872V)

Abbreviations: Mono‐, monoclonal; p‐, phosphorylated; Poly‐, polyclonal; M, Mouse; R, Rabbit.

### 2.4 Cell culture and transfection

Human embryonic kidney cell line (HEK‐293FT), human cervix epithelia cell line (HeLa) and human neuroblastoma SH‐SY5Y cells were cultured in Dulbecco's modified Eagle's medium (DMEM) or DMEM/F12 supplemented with 10% fetal bovine serum (FBS) (Invitrogen, Carlsbad, CA, USA), 100 U/ml penicillin and 100 μg/ml streptomycin, and incubated in a humidified atmosphere containing 5% CO_2_ at 37°C. Cells were seeded to culture plates, and all transfections were performed with FuGENE HD (Promega, Madison, WI, USA) or Lipofectamine™ 2000 (Invitrogen) according to the manufacturer's instructions. Empty vectors were used as controls for the corresponding transfection.

### 2.5 Preparation of various tau fractions from AD brain

Frozen brain tissue samples from autopsied and histopathologically confirmed AD cases were obtained from the Brain Tissue Resource Center, McLean Hospital, Belmont, MA, USA. The use of autopsied frozen human brain tissue was in accordance with the National Institutes of Health guidelines and was exempted by the Institutional Review Board (IRB) of New York State Institute for Basic Research in Developmental Disabilities because “the research does not involve intervention or interaction with the individuals” nor “is the information individually identifiable.”

**FIGURE 1:**
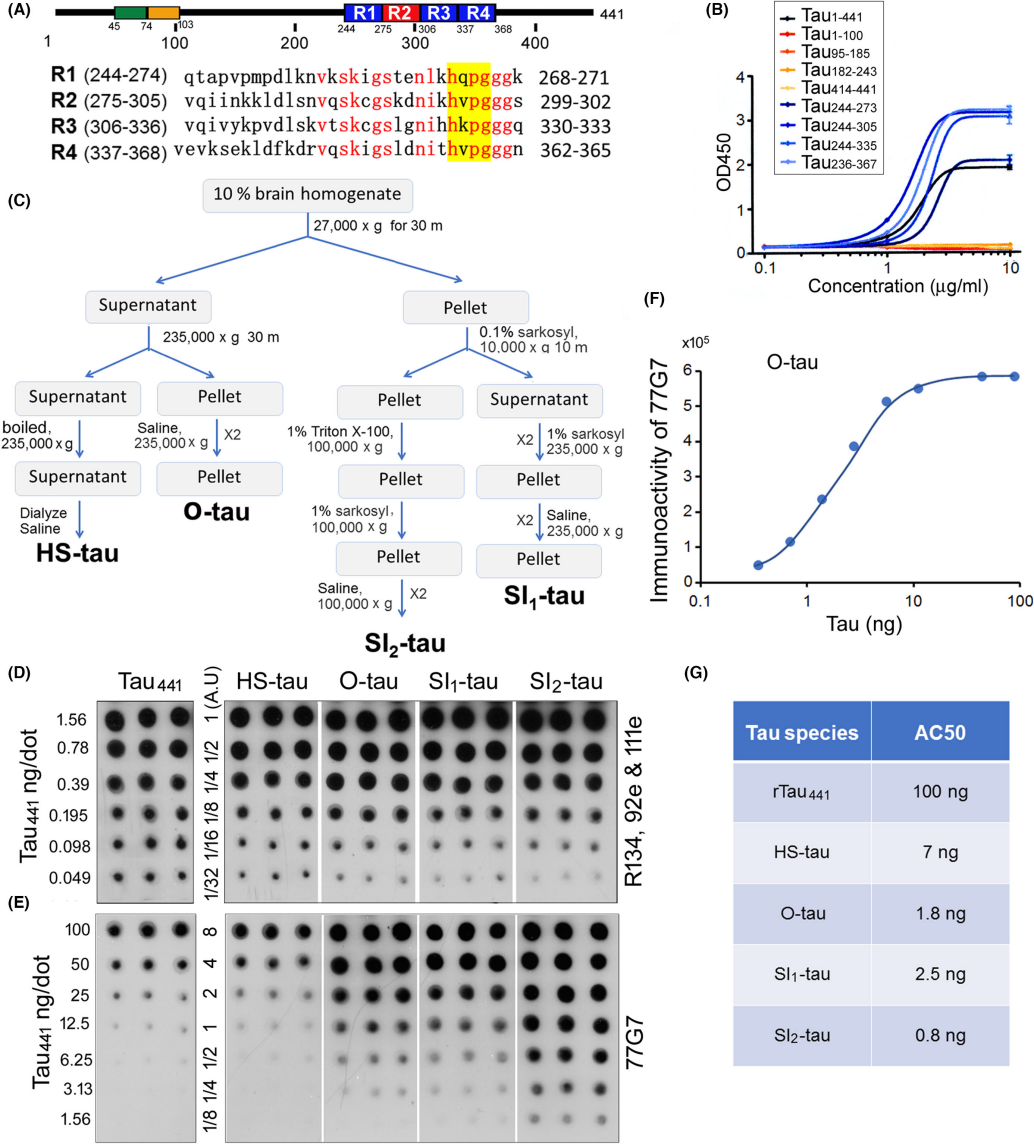
Affinity of 77G7 toward various tau fractions derived from AD brain. (A) By using 15‐mer overlapping synthetic tau peptides, it was found that 77G7 immunoreacted similarly with four peptides (yellow) in four microtubule‐binding repeats of tau (R1‐R4). Homologous amino acid in these four peptides in red. (B) 77G7 reacted with recombinant tau_1–441_ (full‐length), tau_244–273_ (containing R1), tau_244–305_ (containing R1‐R2), tau_244–335_ (containing R1‐R3) and tau_236–367_ (containing R1‐R4) measured by direct ELISA. (C) The main steps used for isolation of various tau fractions from AD brains. (D–F) Various amounts of HS‐tau, AD O‐tau, SI_1_‐tau, and SI_2_‐tau derived from AD brain were dotted on nitrocellulose membrane and developed with the mixture of pan‐tau antibodies R134d, 92e and 111e (D) or 77G7 (E). Blots in Panel B were employed to determine the levels of tau in each tau fractions by using tau_441_ as a standard. 77G7 immunoreactivity of O‐tau were plotted against levels of tau determined by the mixture of R134d, 92e, and 111e in panel B (F). (G) AC50 of various tau fractions detected in panel E

Tau fractions were isolated from autopsied and frozen AD cerebral cortex described previously^37^ by combination of two protocols.^38^, ^39^ Briefly (Figure 1A), 10% brain homogenate prepared in homogenization buffer (20 mM Tris–HCl, pH 8.0, 0.32 M sucrose, 10 mM β‐mercaptoethanol (β‐ME), 5 mM MgSO_4_, 1 mM EDTA, 10 mM glycerophosphate, 1 mM Na_3_VO_4_, 50 mM NaF, 2.0 mM benzamidine, 1.0 mM 4‐(2‐aminoethyl) benzenesulfonyl fluoride hydrochloride (AEBSF), and 10 μg/ml each of aprotinin, leupeptin, and pepstatin) was centrifuged at 27,000 × *g* for 30 min. The pellet was saved for sarkosyl insoluble tau (SI‐tau) preparation. The supernatant was further centrifuged at 235,000 x g for 45 min, and the resulting pellet, i.e., oligomeric tau (O‐tau) enriched fraction, was collected and washed twice with saline and then resuspended in saline. The supernatant was used for heat stable tau (HS‐tau) preparation.

Sarkosyl insoluble aggregated tau preparation: the 27,000 × *g* pellet was homogenized in the homogenization buffer containing 0.1% sarkosyl and centrifuged at 10,000 × *g* for 10 min. The supernatant was adjusted to 1% sarkosyl, incubated for 1 h at room temperature (RT), and then centrifuged at 235,000 × *g* for 45 min. The pellet was washed once with 1% sarkosyl‐homogenization buffer and twice with saline to obtain SI_1_‐tau.

The 10,000 × *g* pellet from above was incubated with 1% Triton X‐100 in homogenization buffer for 30 min at RT and then centrifuged for 1 h at 100,000 × *g*. The resulting pellet was incubated in 1% sarkosyl in homogenization buffer for 1 h at RT and centrifuged at 100,000 × *g* for 30 min. The pellet was washed once with 1% sarkosyl in homogenization buffer and twice with saline and collected as SI_2_‐tau.

Heat stable‐tau (HS‐tau) preparation: the 235,000 × *g* supernatant from above was adjusted to 0.75 M NaCl and 10 mM β‐ME, heated for 5 min at 100°C and then centrifuged at 235,000 × *g* for 30 min. The resulting supernatant was dialyzed against saline to yield HS‐tau.

All fractions were stored at −80°C and sonicated for 10 min at 20% power before used.

### 2.6 Protein expression and purification

The longest human tau isoform tau_441_ and its truncation form, tau_151–391_, were constructed into pGEX‐6p‐1 and expressed in BL21‐Gold *E. Coli* with 0.5 mM IPTG induction for 3 h at RT. The bacteria were lysed by sonication in 50 mM Tris–HCl, pH 7.4, 0.15 M NaCl, 1 mM dithiothreitol (DTT), 1.0 mM Na_3_VO_4_, 50 mM NaF, 1.0 mM AEBSF, and 10 μg/ml each of aprotinin, leupeptin, and pepstatin. Triton X‐100 was added to bacterial lysates to 1% final concentration and centrifuged at 10,000 × *g* for 15 min at 4°C. The supernatant was incubated with glutathione agarose beads for 1 h at 4°C. After extensive washing, the glutathione agarose beads were incubated with PreScission™Protease (Sigma, St. Louis, MO, USA) in buffer (50 mM Tris–HCl, pH 7.0, 150 mM NaCl, 1 mM EDTA, 1 mM DTT) for 16 h at 4°C. Cleaved recombinant tau (rTau) eluted from beads was collected, dialyzed against 10 mM Tris‐buffer (pH 7.4) with 0.5 mM β‐ME, lyophilized, and stored at −80°C until used.

### 2.7 Tau aggregation assay by Thioflavin‐T fluorescence

Recombinant tau (16 μM) was incubated with AD O‐tau (0.35 mg/ml total protein) in 50 μl PBS (phosphate buffered saline) containing 1.13 mM DTT and 33 μM Thioflavin‐T (ThT) in the presence of 2 μM 77G7 or control mouse IgG (mIgG) in 96‐well plate by agitation at room temperature (RT). Fluorescence intensity was measured at various incubation time by Spectrmax M5 at 440 nm excitation and 538 nm emission.

### 2.8 Preparation of fluorescent labeled tau aggregates

Recombinant tau_151–391_ (1 mg/ml) in PBS was incubated with 0.5 mg/ml heparin, 1 mM DTT and a cocktail of proteinase inhibitors (Roche, Basel, Switzerland) at 37°C for 24 h without agitation to induce tau aggregation. The aggregated tau was sonicated for 20 s at 20% amplitude. Tau aggregates were then labeled using Alexa Fluor™ 647 Labeling Kit (Thermo Fisher Scientific, Waltham, MA, USA) according to the manufacturer's instructions. Briefly, tau aggregates were brought to 0.1 M NaHCO_3_ and incubated with Alexa Fluor® dye for 1 h at RT. The fluorescence‐labeled tau aggregates (Tau_agg_‐647) were separated by Sephadex G‐25 column (GE Healthcare Bio‐Sciences, Pittsburgh, PA, USA).

### 2.9 Internalization of tau aggregates

SH‐SY5Y cells were cultured in 8‐well Lab‐Tek II Chamber Glass Slide™ (Nunc, Rochester, NY, USA). Tau_agg_‐647 (150 nM) was incubated with antibody 77G7 (180 nM or 250 nM) for 30 min in cell culture medium and then added to the cell culture. After 4 h culturing, the cells were then washed with PBS for three times, fixed and immunostained with anti‐tubulin followed with secondary fluorescent antibody. The number of internalized tau aggregates or mouse IgG puncta and the total cell numbers were counted from six fields in a well of 8‐well chamber, and the fluorescent puncta were normalized with cell numbers. Three wells for each group were quantified.

### 2.10 Induction of tau_151–391_ aggregation by AD O‐tau in cultured cells

The measurement of tau aggregation induced by AD O‐tau was as described previously.^33^, ^36^ Briefly, cells cultured in 8‐well slides were transfected with pCI/HA‐tau_151–391_ with FuGENE HD. AD O‐tau (1.375 μg total protein) was mixed with 77G7 (1.0 μg or 2.0 μg) and 3% Lipofectamine 2000 in 20 μl of Opti‐MEM and added into wells containing 200 μl culture medium 6 h after transfection. Forty‐two hours after treatment with AD O‐tau, cells were fixed for 15 min with 4% paraformaldehyde (PFA) in PBS and subjected to immunofluorescence staining with anti‐HA. The number of cells with tau aggregates was counted. Average numbers of cells with tau aggregates from 6 fields were normalized with the number of tau expressing cells.

### 2.11 Stereotaxic injection

AD O‐tau was injected bilaterally into the hippocampi of 10‐month‐old female 3xTg‐AD mice or unilaterally into the right hippocampus of 9–11‐month‐old Tg/hTau mice, as described previously.^25^, ^40^ Briefly, mice were deeply anesthetized with 1.25% Avertin (Sigma) and placed in a stereotaxic apparatus. After a craniotomy of 1 mm diameter was performed with a motorized mini‐drill, saline, O‐tau (0.25 μg), or mixture of O‐tau (0.25 μg) with 77G7 (1.5 μg) in 2.38 μl saline was injected into the hippocampus (−2.5 mm anterior/posterior, ±2.0 mm medial/lateral to bregma, and −1.8 mm dorsal/ventral to the dura surface) using a 10 μl Hamilton syringe custom made with a 30‐gauge/0.5 inch/hypodermic needle (Hamilton Syringe Co., Reno, NV, USA) at the rate of 1.19 μl/min for a total of 2 min. For Tg/hTau mice, 0.55 μg O‐tau was injected. The needle was kept in for another 3 min and then was withdrawn slowly to prevent leakage of the infused liquid. The skin was sutured after injection, and the mice were allowed to completely recover on a soft warming pad before being returned to their home cages.

### 2.12 Immunofluorescence staining

3xTg‐AD mice were sacrificed by cervical dislocation 2 months after hippocampal injection. The right hippocampus and the forebrain were quickly dissected and stored at −80°C until used. The left hemisphere was fixed in 4% PFA for 2 days and dehydrated in 30% sucrose solution in PBS. At 10 weeks after injection, the Tg/hTau mice were deeply anesthetized and transcardially perfused with saline followed by buffered 4% PFA. The whole brain was collected, post fixed in the same fixative overnight at 4°C, and dehydrated in buffered 30% sucrose solution. The brains were then cut into 40 μm serial sagittal sections using a freezing microtome, and the free‐floating sections were preserved in antifreeze solution at −20°C till used for immunohistochemical staining.

For immunofluorescence staining, brain sections or cultured cell chamber slides were washed with PBS and treated with 0.3% Triton X‐100 in PBS for 15 min at RT. After blocking with PBS containing 5% normal goat serum, 0.1% Triton X‐100, and 0.05% Tween 20 for 30 min, sections or slices were incubated with primary antibody (Table 1) in the blocking solution overnight at 4°C, washed with PBS, and incubated with Alexa 488 or Alexa 555‐conjugated‐secondary antibody for 2 h at RT. TO‐PRO‐3 iodide (5 mg/ml) was used to stain nuclei. After washing with PBS, sections were mounted on microscopic slides with ProLong™ Gold antifade reagent (ThermoFisher Scientific) and set under a coverslip before imaging on a Nikon confocal microscope. For quantification, average numbers of AT8 or T22 positive neurons in the hippocampus were quantified from 3–5 brain sections from the left hemisphere per mouse.

### 2.13 Western blots

Brain tissue was homogenized in cold buffer (50 mM Tris–HCl, pH 7.4, 150 mM NaCl, 10 mM β‐ME, 1 mM EDTA, 1 mM Na_3_VO_4_, 50 mM NaF, 1.0 mM AEBSF, and 10 μg/ml each of aprotinin, leupeptin, and pepstatin) (1:9 w/v). Brain homogenates were adjusted to 1× Laemmli sample buffer, followed by heating in boiling water‐bath for 5 min. Cultured cells were lysed directly in Laemmli sample buffer and then heated as above. Protein concentration was determined using the Pierce™ 660 nm Protein Assay kit (ThermoFisher Scientific). Samples were subjected to SDS‐PAGE and transferred onto polyvinylidene fluoride membrane (MilliporeSigma, Burlington, MA). The Membrane was subsequently blocked with 5% fat‐free milk in TBS (Tris‐buffered saline) for 30 min, incubated with primary antibody (Table 1) diluted in 5% fat‐free milk in TBS containing 0.1% NaN_3_ overnight, washed with TBST (TBS with 0.05% Tween 20) for three times, and incubated with HRP conjugated secondary antibody for 2 h at RT, washed with TBST, incubated with the enhanced chemiluminescence (ECL) substrate (ThermoFisher Scientific), and exposed to HyBlot CL® autoradiography film (Denville Scientific Inc., Holliston, MA). Specific immunoblotting was quantified by using the Multi Gauge software V3.0 from Fuji Film (Minato, Tokyo, Japan).

### 2.14 Immuno‐dot‐blot and capture assays

HEK‐293FT cells transfected with pCI/HA‐tau_151–391_ were probe sonicated in cold PBS containing 1.0 mM AEBSF and 10 μg/ml each of aprotinin, leupeptin, and pepstatin for 2 min with 20% amplitude at 4°C at 48 h after transfection. Cellular extract, extra/tau_151–391_, was stored at −80°C until used.

Various amounts of O‐tau were dotted onto nitrocellulose membrane (Schleicher and Schuell, Keene, NH, USA) at 5 μl per grid of 7 × 7 mm size in triplicates. The membrane was placed in a 37°C oven for 1 h to allow the protein to bind to the membrane. For immuno‐dot blot assay, the membrane was subsequently blocked, incubated with primary antibody and then secondary antibody as described above for Western blots.

For capture assay, the membrane was blocked and incubated with above extra/tau_151–391_ in the presence of 77G7 (0.285 or 0.57 μM) overnight. After washing, the membrane was incubated with anti‐HA followed by incubation with secondary antibody and ECL as described above to detect bound tau.

### 2.15 Statistical analysis

One‐way or two‐way analysis of variance (ANOVA) followed by Tukey's test or Sidak's multiple comparisons test was used in this study. Comparison between two groups was analyzed by unpaired two‐tailed Student's *t*‐test. Shapiro–Wilk test and/or Kolmogorov–Smirnov test for normality were used to assess data distribution. Data were presented as mean ± standard deviation (SD) or mean ± standard error (SEM) as indicated and analyzed using GraphPad Prism 6 (GraphPad Software Inc., San Diego, CA, USA). *p* < 0.05 was considered statistically significant.

## 3 RESULTS

### 3.1 77G7 displays higher affinity toward aggregated than non‐aggregated tau fractions derived from AD brain

Monoclonal tau antibody 77G7 was generated in our laboratory by using recombinant human tau_441_. By using overlapping synthetic tau peptides, we found that 77G7 reacted with the tau peptides containing “HQPG” within microtubule‐binding repeat 1 (R1), “HVPG” within R2, “HKPG” within R3, and HVPG within R4 (Figure 1A), suggesting four discontinuous epitopes crossing microtubule‐binding repeats. Comparing the sequence, we found that these four epitopes contain “HxPG”. Furthermore, we found that by using recombinant tau fragments, 77G7 reacted with Tau_1–441_, tau_244–273_ (R1), tau_244–305_ (R1‐R2), tau_244–335_ (R1‐R3), and tau_236–367_ (R1‐R4), but not tau_1–243_ and tau_414–441_ (Figure 1B), confirming that 77G7 targets the microtubule‐binding repeats of tau.

In addition to hyperphosphorylated and aggregated tau, oligomeric species and filaments, AD brain contains similar level of un‐aggregated tau as in age‐matched normal brain,^2^, ^38^, ^41^, ^42^ which is cytosolic and heat‐stable.^33^, ^43^ To determine whether 77G7 reacts with the aggregated tau and un‐aggregated tau similarly, we separated tau from AD brain into different fractions, heat‐stable un‐aggregated tau (HS‐tau), oligomeric‐tau (O‐tau), sarkosyl insoluble 1 tau (SI_1_‐tau), sarkosyl insoluble 2 tau (SI_2_‐tau) (Figure 1C) and analyzed the immunoreactivity of 77G7 toward them. We first determined tau levels in the preparations of these AD tau fractions by immuno‐dot blots developed with a mixture of pan‐tau rabbit polyclonal antibodies, R134d, 92e, and 111e, using recombinant tau_441_ (rTau_441_) as a standard (Figure 1D). Then, we analyzed immunoreactivity of 77G7 toward each of the AD tau fractions (Figure 1E) and plotted the immunoreactivity of 77G7 against the levels of tau in O‐tau (Figure 1F). The amount of tau reached half maximal immunoreactivity (AC_50_) was used to assess the affinity to 77G7. The AC_50_s of rTau_441_, HS‐tau, O‐tau, SI_1_‐tau, or SI_2_‐tau were 100, 7, 1.8, 2.5, and 0.8 ng, respectively (Figure 1G). These results suggest that 77G7 antibody displays much higher affinity toward aggregated tau pools, O‐tau, SI_1_‐tau, and SI_2_‐tau, than un‐aggregated HS‐tau derived from AD brain; rTau_441_ had the lowest affinity to 77G7.

### 3.2 77G7 suppresses the entry of tau aggregates into SH‐SY5Y cells

**FIGURE 2:**
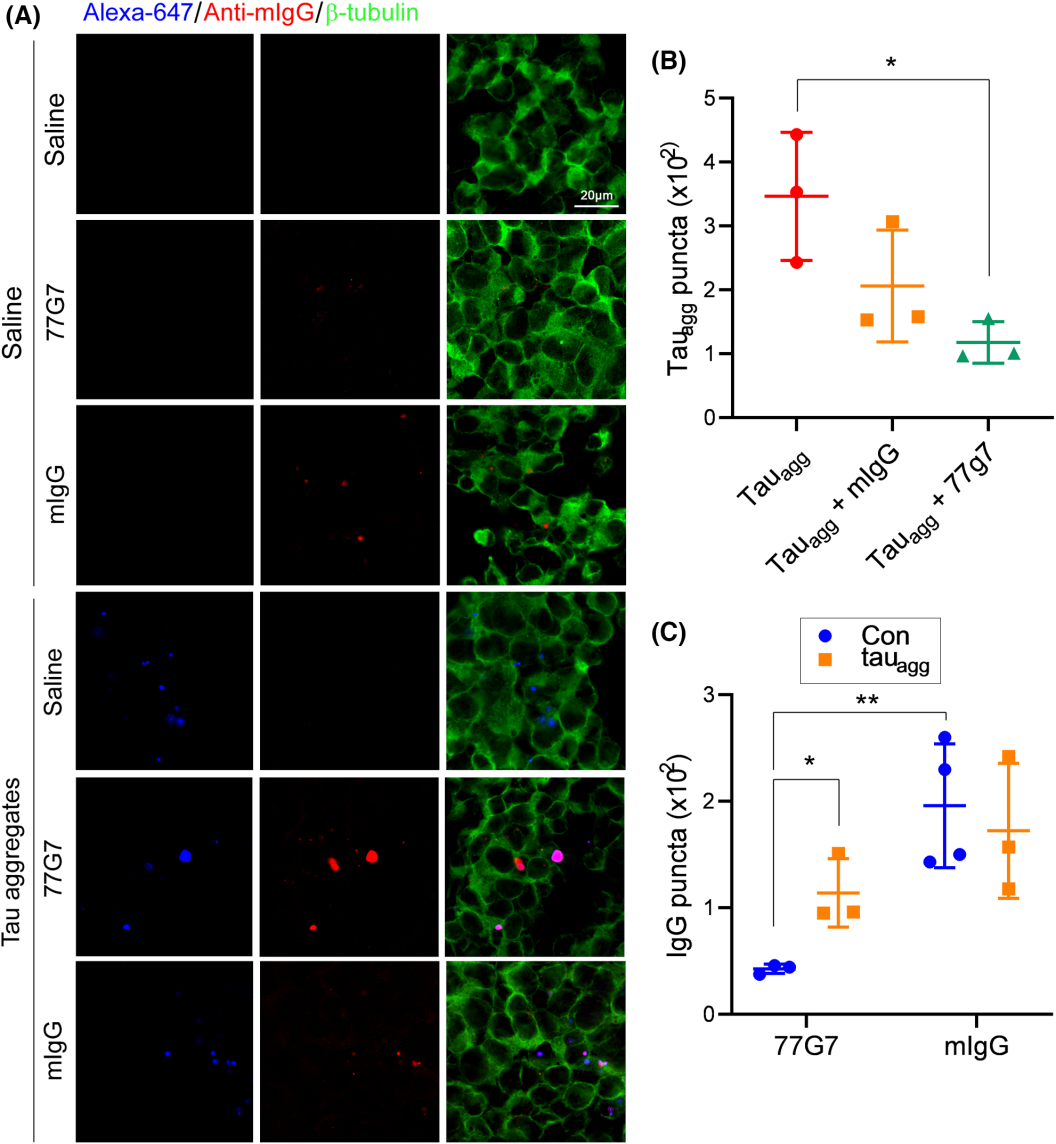
Effect of 77G7 on the internalization of tau aggregates. (A) SH‐SY5Y cells were treated with Tau_agg_‐647 (150 nM in tau) pre‐incubated with 77G7 (180 nM) or mouse IgG (mIgG, 250 nM) for 4 h. The cells were immunostained with anti‐tubulin (green) and anti‐mouse IgG (red) antibodies. Tau_agg_‐647 is displayed in blue. (B,C) The numbers of Tau_agg_‐647 and antibody puncta were counted and are presented in scattered dots with mean ± SD (*n* = 3–4). Shapiro–Wilk test for normality was used to assess data distribution. One‐way ANOVA or two‐way ANOVA followed by Tukey's test or Sidak's multiple comparisons test was used for statistical analyses. *p < 0.05; **p < 0.01

To learn whether 77G7 influences internalization of tau aggregates, we generated tau_151–391_ aggregates in vitro with heparin and labeled the aggregates with Alexa fluor 647 (Tau_agg_‐647). Then, we incubated Tau_agg_‐647 (150 nM tau) with 77G7 antibody (180 nM) or mouse IgG (mIgG) (250 nM) for 30 min at RT and added them to the culture medium of SH‐SY5Y cells. After 4 h culture, the cells were immunostained with anti‐mouse IgG and anti‐tubulin. Tau_agg_‐647 (Figure 2A, blue), IgG (Figure 2A, red), and tubulin (Figure 2A, green) were visualized by confocal microscopy. We found puncta of Tau_agg_‐647 were colocalized well with 77G7 and partially with mIgG in cells (Figure 2A). Compared with vehicle treatment, the number of Tau_agg_‐647 in the SH‐SY5Y cells was less with 77G7 antibody pre‐incubation (Figure 2B), suggesting that 77G7 suppresses the entry of tau aggregates into the SH‐SY5Y cells. A higher concentration of mIgG (250 nM) did not significantly affect the internalization of Tau_agg_‐647 (Figure 2B). Interestingly, by quantifying the number of immunostained mouse IgG puncta, we found that internalized 77G7 were increased by pre‐incubation of the antibody with Tau_agg_‐647 (Figure 2C), indicating that tau aggregates may facilitate the entry of 77G7 into cells. Tau_agg_‐647 did not influence mIgG internalization.

### 3.3 77G7 inhibits the seeding activity of AD O‐tau in vitro and in cultured cells

**FIGURE 3:**
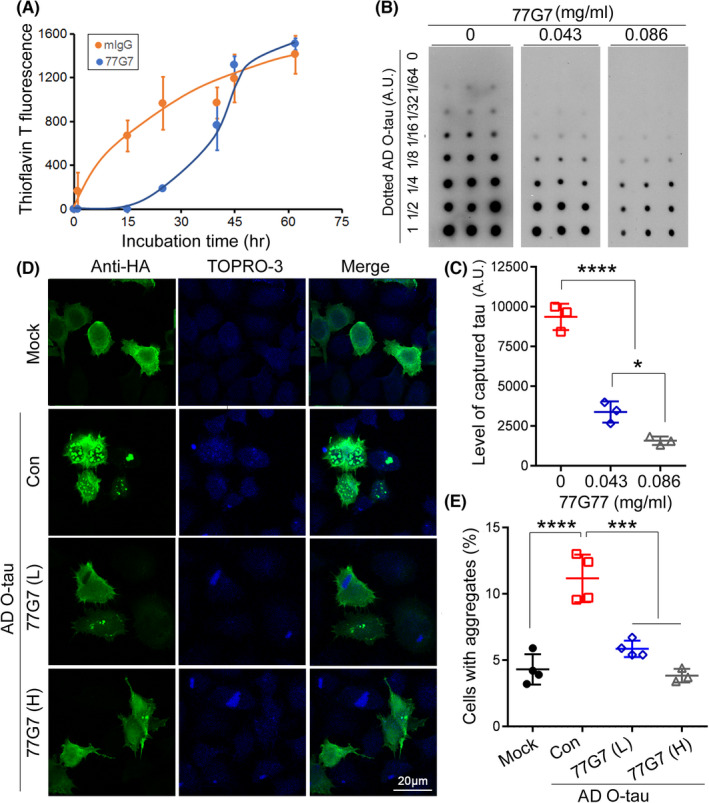
Effect of 77G7 on the seeding activity of AD O‐tau. (A) Recombinant Tau_151–391_ was incubated with AD O‐tau to induce the aggregation in the presence of 77G7 or mIgG together with Thioflavin T at room temperature. The fluorescence density was determined at various time points and plotted against the incubation time to assay the aggregation of tau. (B,C) Various amounts of AD O‐tau were dotted onto nitrocellulose membrane and incubated with HA‐tau_151–391_ containing cell extract overnight at room temperature in the presence or, as a control, absence of 77G7 (0.043 mg/ml or 0.086 mg/ml). Captured tau was analyzed by anti‐HA followed by HRP‐anti‐mouse IgG and ECL. (D,E) HeLa cells were overexpressed with HA‐tau_151–391_ and then treated with AD O‐tau in the presence or, as a control, absence of 77G7 (30 nM [L] or 60 nM [H]) for 48 h. The cells were immunostained with anti‐HA (D). TO‐PRO‐3 was used for nuclear staining. The percentage of cells with tau aggregates in tau expressed cells was assessed (E) and are presented in scattered plots with mean ± SD. Shapiro–Wilk test for normality were used to assess data distribution. One‐way ANOVA followed by Tukey's multiple comparisons test was used for statistical analyses. *p < 0.05; ***p < 0.001; ****p < 0.0001

Alzheimer's disease O‐tau captures tau and templates tau aggregation in vitro and in vivo.^25^, ^33^, ^40^, ^44^ To determine the effect of 77G7 on AD O‐tau induced tau aggregation in vitro, we incubated AD O‐tau with Tau_151–391_ in the presence of 77G7 or control mIgG and monitored tau aggregation by Thioflavin T (ThT) fluorescence. We found that AD O‐tau induced tau aggregation as indicated by increase in fluorescence within 72 h in the presence mIgG (Figure 3A). However, 77G7 suppressed AD O‐tau‐induced‐tau aggregation at the initial phase (0–15 h), but tau aggregates formed quickly at the next stage (15–48 h) and gradually reached the maximum at the end stage (48–72 h) with similar level of fluorescence intensity with mIgG group (Figure 3A). We speculate that long time incubation at room temperature may inactivate 77G7, leading to insufficient inhibition of AD O‐tau‐induced tau aggregation at late phase. These results suggest that 77G7 inhibits and delays tau aggregation induced by O‐tau in vitro.

To determine the effect of 77G7 on AD O‐tau to recruit tau, we performed tau capture assay. We overexpressed HA‐tau_151–391_ in HEK‐293FT cells (HEK‐293/HA‐Tau_151–391_) and lysed the cells in PBS containing the cocktail of the inhibitors of proteinases and phosphatases by probe‐sonication. Meanwhile, various amounts of AD O‐tau were applied on nitrocellulose membrane, and then the membrane was incubated with the crude extract of HEK‐293/HA‐Tau_151–391_ in the presence of 0.043 mg/ml or 0.086 mg/ml 77G7. The captured HA‐Tau_151–391_ from the cell extract was analyzed with anti‐HA. We found that the captured tau levels were reduced in the presence of 77G7 and less tau was captured by high dose of 77G7 (Figure 3B,C). Thus, 77G7 suppresses AD O‐tau's activity to capture soluble tau.

To study whether 77G7 inhibits AD O‐tau‐seeded tau aggregation in cultured cells, we overexpressed HA‐Tau_151–391_ in HeLa cells and treated the cells with AD O‐tau pre‐incubated with or without 77G7. Tau aggregates were visualized by immunofluorescent staining with anti‐HA 42 h after AD O‐tau treatment. We observed 12% Tau_151–391_‐expressing cells with tau aggregates by AD O‐tau treatment, but under baseline control condition, <4% cells formed tau aggregates (Figure 3D,E). The percentage of cells with tau aggregates was significantly reduced when they were treated with pre‐incubated AD O‐tau with 30 nM 77G7 (Figure 3E). A smaller number of cells with tau aggregates were observed when treated with a higher dose of 77G7 (60 nM, Figure 3E). Thus, 77G7 effectively blocks AD O‐tau to seed tau aggregation in cultured cells.

### 3.4 77G7 inhibits AD O‐tau to seed tau pathology in vivo

We previously reported that AD O‐tau can seed tau pathology in vivo.^25^, ^29^, ^40^ To study the effect of 77G7 on AD O‐tau induced tau aggregation in vivo, we bilaterally injected 0.25 μg AD O‐tau or a mixture of 0.25 μg AD O‐tau with 1.5 μg 77G7 or with mIgG into the hippocampi of 10‐month‐old 3xTg‐AD mice, a widely used AD mouse model.^45^ Control mice were injected with same volume of saline. The left and right hemispheres were collected for biochemical and immunohistochemical studies, respectively, 2 months after the injection.

**FIGURE 4:**
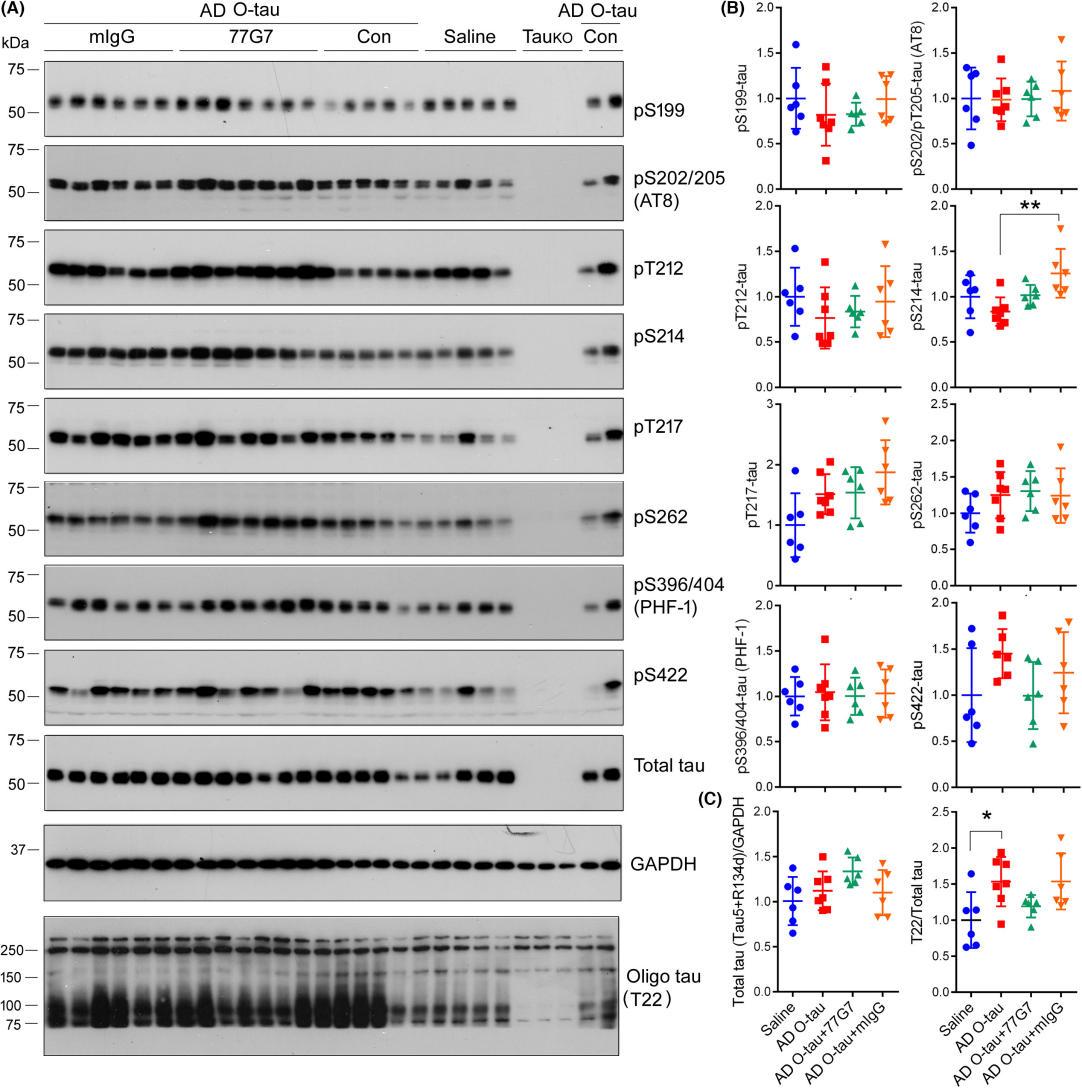
Effect of 77G7 on the level of tau phosphorylation in the hippocampus injected with AD O‐tau. (A) 3xTg‐AD mice (10‐month‐old) were bilaterally injected with AD O‐tau or mixture of AD O‐tau with 77G7 or mIgG into the hippocampus. Two months later, left hippocampus was used for analysis of tau hyperphosphorylation and oligomerization by Western blots developed with the indicated antibodies. Samples from tau knockout mice (Tau_KO_) were included to verify the specificities of the tau antibodies. The 150 kDa beyond T22 immunoreactive bands were not quantified since they were also present in Tau_KO_ mouse hippocampus. (B,C) Levels of phosphorylated tau, total tau or oligomeric tau (Oligo tau, T22) were normalized with total tau or GAPDH and are presented as scattered plots with mean ± SD. Shapiro–Wilk test and Kolmogorov–Smirnov test for normality were used to assess data distribution. One‐way ANOVA followed by Tukey's test was used for statistical analyses. *p < 0.05; **p < 0.01

To study the effect of 77G7 on tau phosphorylation in vivo, the left hippocampi were analyzed by Western blots developed with phosphorylation‐dependent and site‐specific tau antibodies. We did not find any significant alteration of tau phosphorylation at Ser199, Ser202/Thr205 (AT8), Thr212, Ser214, Thr217, Ser262, Ser396/404 (PHF‐1), and Ser422 in AD O‐tau‐injected hippocampi by Western blots (Figure 4). No immunoreactivity in Tau_KO_ mouse hippocampi indicated the specificity of these antibodies toward tau. Tau phosphorylation at these sites was not altered significantly in the hippocampi injected with mixture of AD O‐tau with 77G7 or mIgG either (Figure 4). Thus, these results suggest that 77G7 could not influence tau phosphorylation significantly in injected hippocampus by injecting a mixture of it with AD O‐tau.

Then, we analyzed oligomeric tau level by Western blots developed with T22, a polyclonal antibody to oligomeric tau.^46^ We found that AD O‐tau injection increased hippocampal immunoreactivity to T22 significantly (Figure 4). Compared with AD O‐tau injection alone, T22 immunoreactivity was clearly reduced in mouse hippocampus with injection of mixture of AD O‐tau with 77G7, but not with mIgG (Figure 4). Tau_KO_ mouse hippocampi were employed as a negative control to determine the specificity of T22 toward tau. These results suggest that 77G7 suppresses AD O‐tau to seed tau aggregation in vivo in mouse brain.

**FIGURE 5:**
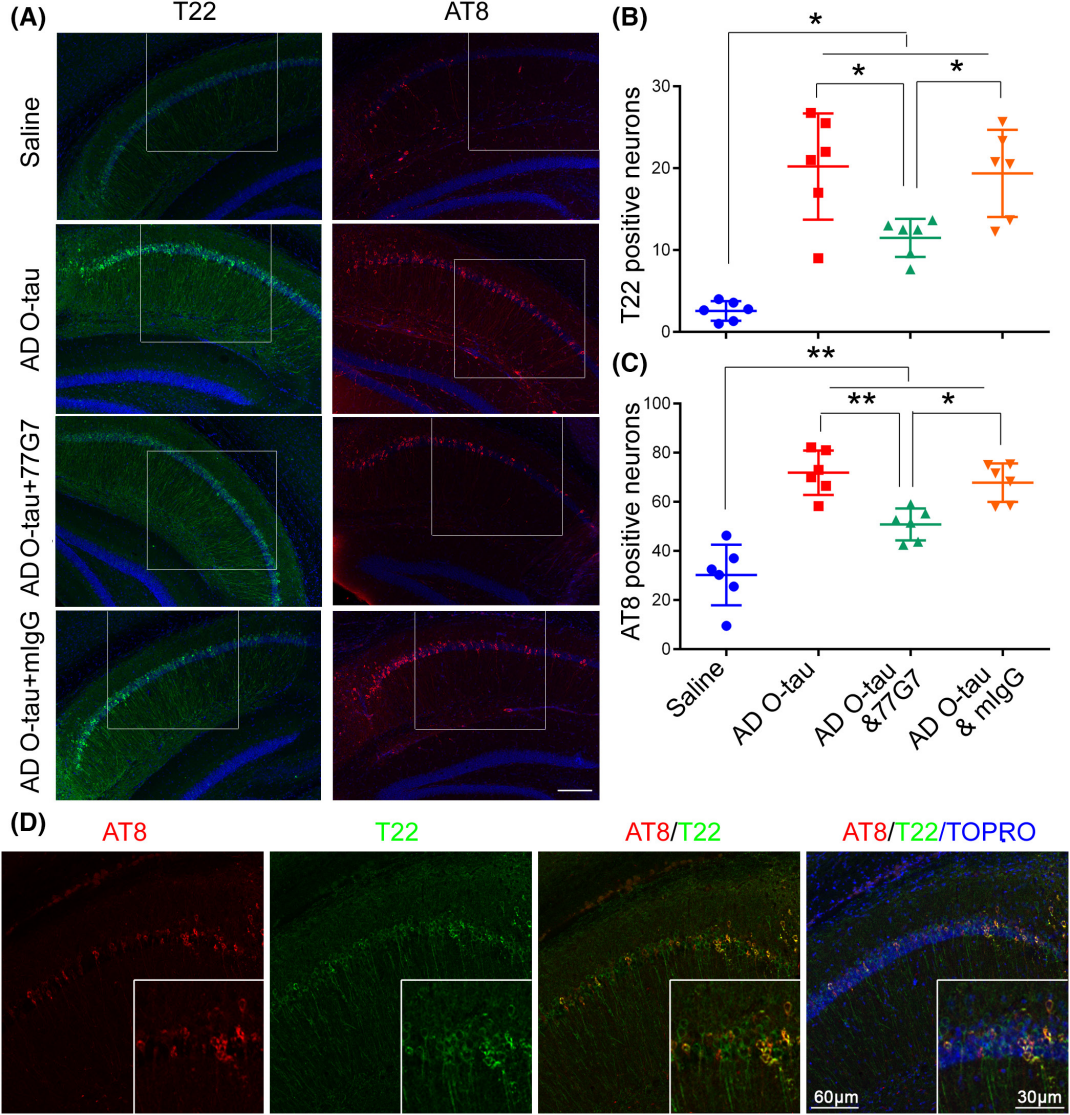
Effect of 77G7 on AD O‐tau‐induced tau pathology in mice. (A–D) Sagittal sections of AD O‐tau injected 3xTg‐AD mouse brains were immunostained with T22 (oligomeric tau) or AT8 (phospho‐tau at Ser202/205) (A). The numbers of T22 (B) or AT8 (C) positive cells in CA1 region were counted and average positive cell numbers from 3 to 5 brain sections per mice were calculated. Shapiro–Wilk test and Kolmogorov–Smirnov test for normality were used to assess data distribution. Data were statistically analyzed with one‐way ANOVA with post‐Tukey's multiple comparisons test and are presented as scattered plots with mean ± SD. *p < 0.05; **p < 0.01. (D) Double‐immunostaining of the brain section from AD O‐tau‐injected mice with T22 (green) and AT8 (red) antibodies. TOPRO‐3 was used for nuclear staining

Tau Oligomer detected by T22 is regarded as early stages of tau pathology in AD,^46^ whereas AT8 antibody is widely used to stain pre‐tangles and neurofibrillary tangles in immunohistochemical studies.^10^, ^47^ We immunostained the right brain sagittal sections with T22 and AT8 antibodies and counted the immuno‐positive cell numbers in CA1 region of the 3–5 sagittal sections per brains. We found that AD O‐tau injection resulted in a marked increase in the number of T22 and AT8 positive neurons, while they were significantly decreased in the mice injected with mixture of AD O‐tau and 77G7 (Figure 5A–C), but not in the control mIgG mice (Figure 5A–C). These results suggest that 77G7 inhibits AD O‐tau to seed tau aggregation in 3xTg‐AD mouse hippocampi.

The double‐immunostaining of the brain section from AD O‐tau injected mice with T22 and AT8 antibodies showed that only large aggregates were both T22 and AT8 positive, while the small aggregates stained with T22 but not with AT8 (Figure 5D), supporting that during tau pathogenesis, T22 pathological change may precede AT8 pathological change.^46^

### 3.5 77G7 suppresses site‐specific tau phosphorylation induced by O‐tau in Tg/hTau mice

**FIGURE 6:**
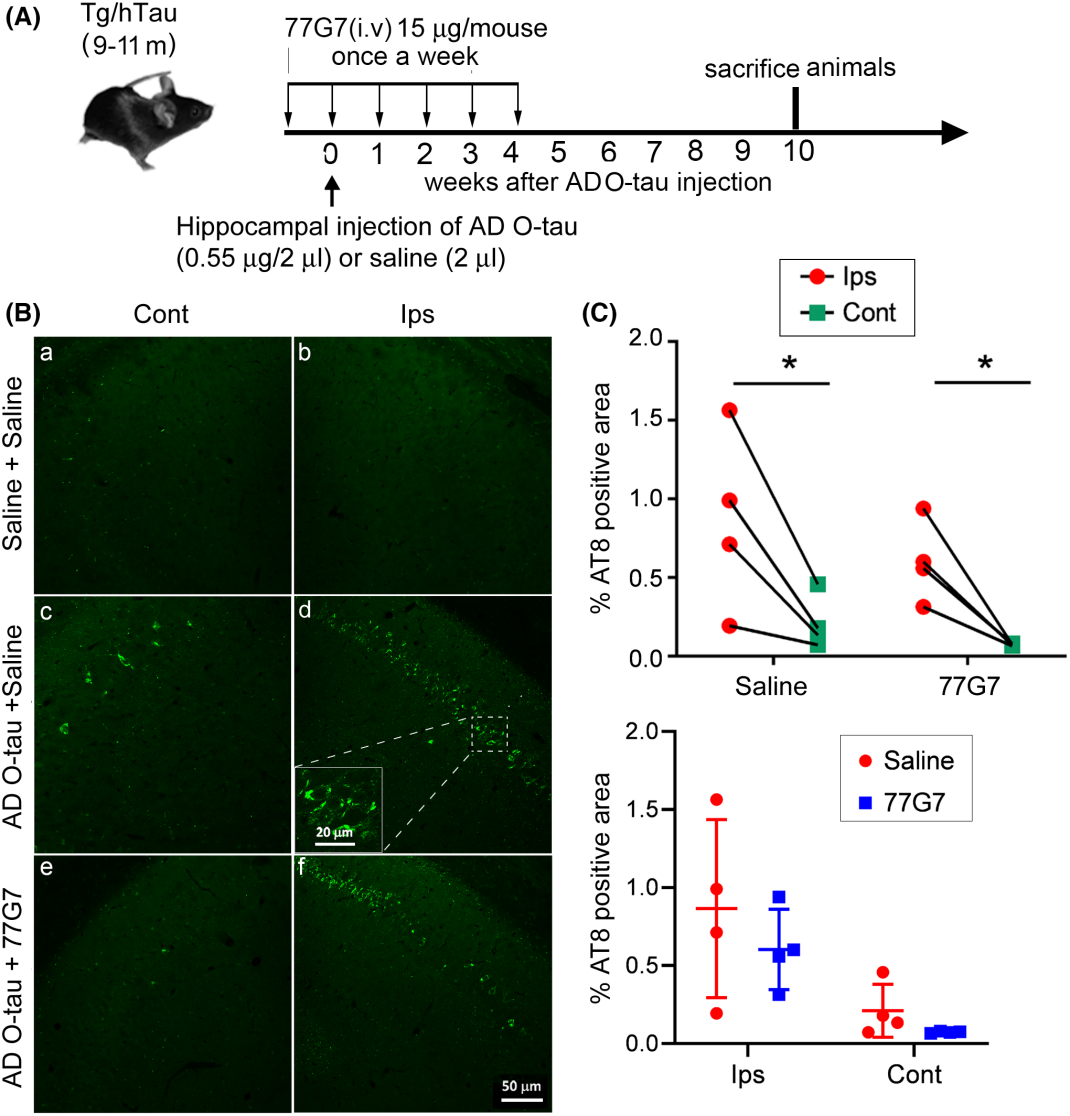
Role of 77G7 intravenous administration in tau pathology induced by AD O‐tau in Tg/hTau mice. (A) The experimental design. Tg/hTau mice were immunized with 77G7 (iv, 15 μg in 200 μl saline) once a week for 6 weeks. AD O‐tau (0.55 μg in 2 μl saline) was unilaterally injected into the hippocampus on the same day of the second dose of immunization. All animals were sacrificed at the 10th week post‐AD O‐tau injection. Brains were collected for immunohistochemical and biochemical studies. (B,C) Coronal brain sections were immunostained with tau antibody AT8. Immunostaining of both the ipsilateral (Ips) and contralateral (Cont) hippocampi of Tg/hTau mice were captured and quantified with ImageJ software. Shapiro–Wilk test for normality was used to assess data distribution. Data were statistically analyzed with paired two‐way ANOVA with post‐Sidak's multiple comparisons test and are presented as scattered plots with mean ± SD (C). *p < 0.05. Scale bar 50 μm and insert scale bar 20 μm

Above results suggest that 77G7 may effectively inhibit AD O‐tau seeding activity in vitro and in vivo (see Figures 3, 4, 5). To learn whether peripheral 77G7 administration can inhibit AD O‐tau induced tau pathology, we intravenously injected 15 μg 77G7 in 200 μl saline 1 week before the unilateral injection of 0.55 μg AD O‐tau into the hippocampus of 9‐11‐month‐old Tg/hTau mice and followed by weekly iv‐injection of 77G7 for 5 weeks (Figure 6A). Saline was intravenously or hippocampally injected as respective controls. We analyzed tau pathology by AT8 immunostaining and tau phosphorylation by Western blots 6 weeks after antibodies injection. We found that no AT8 positive cells in saline injected hippocampi, suggesting no tau pathology in the hippocampus of 12–14‐month‐old Tg/hTau mouse (Figure 6B), but a robust tau pathology was detected by AT8 staining in the ipsilateral and contralateral hippocampi of AD O‐tau injected mice (Figure 6B). Tau pathology in the contralateral hippocampi was much less than that in the ipsilateral sites (Figure 6B,C). AT8 immunoactivity was less but did not reach statistical significance in both ipsilateral and contralateral hippocampi of 77G7 iv‐injected mice (Figure 6B,C).

**FIGURE 7:**
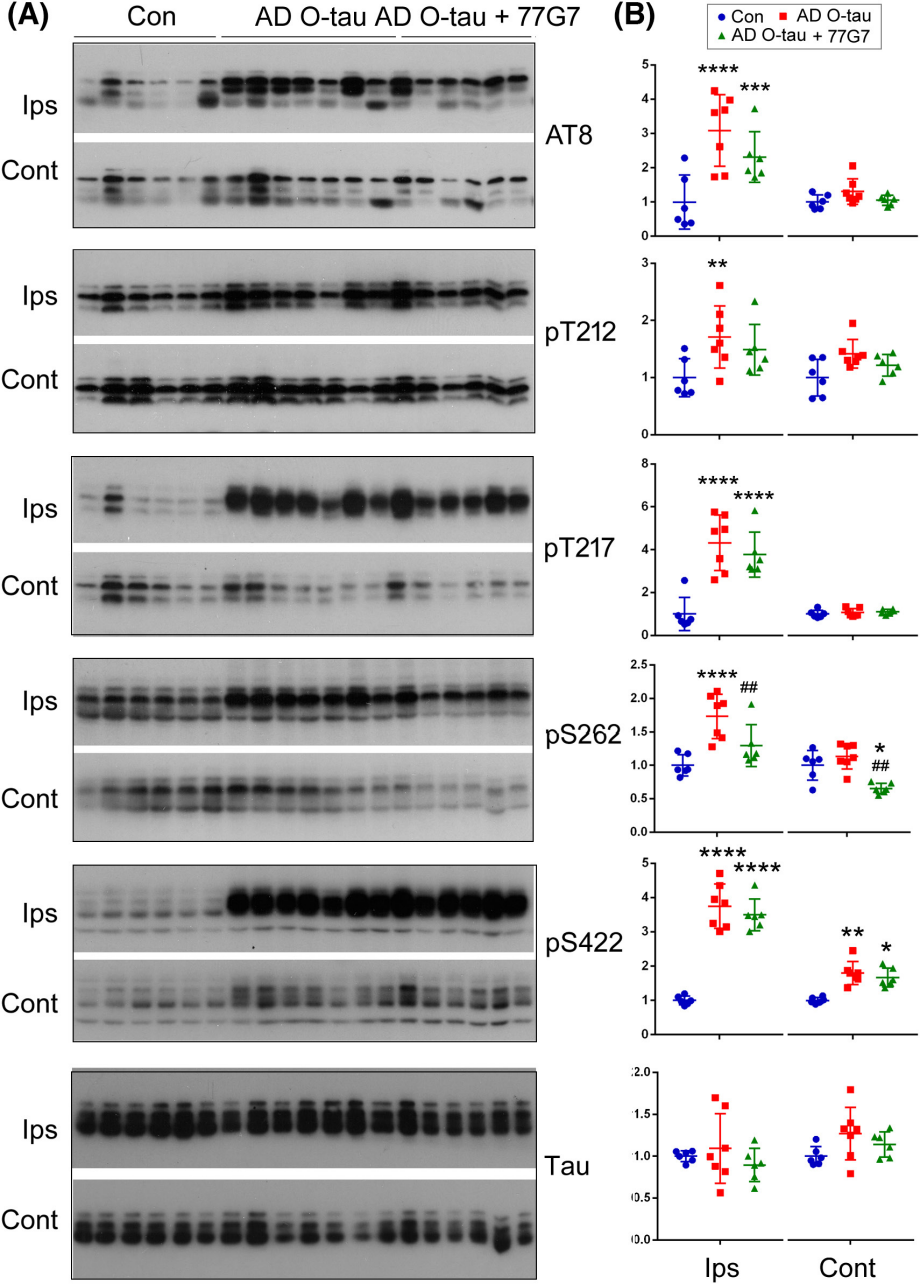
Effect of immunization with 77G7 on hyperphosphorylation of tau induced by AD O‐tau. (A) Tg/hTau mice were unilaterally injected with AD O‐tau and treated with 77G7 or saline as described in Figure 6A. Tau phosphorylation in ipsilateral and contralateral hippocampi was analyzed by Western blots developed with site‐specific and phosphorylation‐dependent tau antibodies. (B) Levels of phospho‐tau are presented as scattered plots with mean ± SD. Shapiro–Wilk test and Kolmogorov–Smirnov test for normality were used to assess data distribution. Data were statistically analyzed with one‐way ANOVA with post Tukey's multiple comparisons test. *p < 0.05; **/^##^ *p* < 0.01; ***p < 0.001; *p* < 0.0001. *, vs Control; #, 77G7 vs AD O‐tau treatment

We previously reported that AD O‐tau induced tau hyperphosphorylation in site specific manner.^40^ To determine the effect of 77G7 iv‐injection on tau phosphorylation induced by AD O‐tau, the ipsilateral and contralateral hippocampi were analyzed by Western blots. We found that AD O‐tau induced hyperphosphorylation of tau in site‐specific manner (Figure 7A), which was attenuated at Ser262 or showed a reduction trend at Ser202/Thr205 (AT8 sites), Thr212, Thr217, and Ser422 in both hippocampi of mice with 77G7 iv‐injection (Figure 7A,B). Thus, immunotherapy with 77G7 can ameliorate hyperphosphorylation of tau induced by AD O‐tau in vivo.

## 4 DISCUSSION

Number of NFTs, the hyperphosphorylated tau aggregates, correlates positively with dementia in AD and its stereotypical distribution associates with the progression of AD, making tau an attractive target for the therapy of AD and related tauopathies. Tau immunotherapies have shown promise in pre‐clinical and clinical studies.^26^ The present study reveals that tau antibody 77G7 binds to the four highly homologous and yet independent tetrapeptides located in each microtubule‐binding repeat (MTBR) and has a high affinity to aggregated tau and not un‐aggregated tau derived from AD brain, inhibits the cellular uptake of tau aggregates and suppresses the seeding activity of AD O‐tau to recruit tau and to template tau aggregation in vitro and in vivo in tau transgenic mice. Intravenous administration of 77G7 ameliorated AD O‐tau‐induced‐hyperphosphorylation of tau in site‐specific manner. Taken together, these findings suggest that 77G7 may have therapeutic potential to inhibit the propagation of tau pathology.

Passive and active immunization against phosphorylated tau, the N‐terminal region, middle region, microtubule binding region and C‐terminal region have shown various degrees of efficacy in reducing tau pathology and improving cognitive or motor functions in tau transgenic mice.^48^ Microtubule binding repeats not only play critical role in polymerization and stability of microtubule^49^ but also are responsible for the pathological aggregation of tau.^50^, ^51^, ^52^ Tau in AD brain is truncated at multiple sites^53^ and SDS‐ and reducing‐regent‐resistant high molecular tau lacks N‐terminal domain.^54^ Of special note, oligomeric tau, the most potent tau seeds, in AD brain highly immunoreacts with antibodies targeting MTBR, but not N‐terminal portion of tau.^37^ Thus, it is reasonable to speculate that antibodies against MTBR may inhibit seeding and spreading of tau with high efficiency. However, only two preclinical studies on tau passive immunotherapies targeting MTBR are carrying out.^27^, ^55^ The epitope of antibody HJ9.3 is a.a. 306–321^27^ and antibody DC8E8 is sufficient to recognize the 6‐mer peptides with consensus sequence HXPGGG localized in the MTBR region of tau.^55^ Both antibodies block uptake and seeding activity of tau aggregates and are effective in reducing tau pathology in transgenic rodent models. 77G7 is a monoclonal antibody generated by using recombinant tau_441_ as an immunogen. Of interesting note, we found here that 77G7 immunoreacted with the peptides containing HXPG localized in each of the four microtubule binding repeats. Thus, similar to DC8E8, 77G7 binds four highly homologous and independent epitopes in MTVRs. It was reported that DC8E8 is highly discriminatory between pathological and physiological tau.^55^ We also found here that 77G7 has higher affinity toward aggregated tau (O‐tau, SI_1_‐tau and SI_2_‐tau) than un‐aggregated tau (HS‐tau and rTau_441_). 77G7 effectively delayed AD O‐tau‐induced tau aggregation in vitro and in vivo, suppressed AD O‐tau's ability to capture tau from cell lysate, and to inhibit AD O‐tau seeded tau aggregation in cultured cells and in mice, suggesting that 77G7 is sufficient to inhibit the seeding activity of O‐tau. Thus, 77G7 may serve a preferred antibody for immunotherapy to target pathological tau effectively and specifically.

The propagation of tau pathology involves the release of proteopathic tau aggregates from donor cells into extracellular space and then uptake by neighboring cells. Tau can be released into the extracellular space by an unconventional protein secretion mechanism through direct translocation across cell membrane mediated by sulfated proteoglycans,^56^ exosome,^57^, ^58^ ectosomes,^59^ and by nanotubes.^60^ Several models were proposed to elucidate the mechanism of entry of tau aggregates into neurons. Some believed that tau aggregates bind to heparan sulfate proteoglycans (HSPGs) to stimulate cell uptake via macropinocytosis in a clathrin‐independent endocytosis manner and seed further aggregation.^61^, ^62^ Another opinion is that aggregated tau enters into neuron primarily through dynamin‐dependent endocytosis.^63^ Studies have showed that specific tau antibody could interrupt the spread of tau pathology by binding to tau fibrils and consequently inhibiting their entry into neurons.^28^, ^63^ Here, we found that 77G7 suppressed uptake of tau aggregates by SH‐SY5Y cells. Moreover, an increase in the entry of 77G7 was found in cells treated with the mixture of antibody and O‐tau, indicating that O‐tau may also help 77G7 enter the cells by forming antibody–antigen complex. Monoclonal tau antibodies were reported to be uptaken by neurons through receptor‐mediated endocytosis and the intracellular endosome/autophagosome/lysosome system were likely involved in antibody‐mediated clearance of pathological Tau.^64^

Cell models were developed to study the seeding activity of proteopathic tau aggregates from AD brain based on tau transmission between cell to cell.^33^, ^65^ After entry into cells, tau aggregates recruit soluble tau monomers and template them to form insoluble tau aggregates. HEK‐293 TauRD‐P301S‐CFP/YFP expressing biosensor cells were used to detect seeding activity of tau seeds from brains^65^ or generated in vitro.^66^ By using the biosensor cells, it was reported that the earliest and most robust seeding activity is in the transentorhinal/entorhinal cortices of AD and that tau aggregates precede overt tau pathology.^67^, ^68^ In the present study, we found tau aggregation in HeLa/Tau_151–391_ cells treated with AD O‐tau. By using the cell model, we found 77G7 significantly suppressed AD O‐tau seeded tau aggregates and high dose of 77G7 almost completely blocked AD O‐tau induced tau aggregation.

77G7 was also found to reduce tau pathology induced by hippocampal injection with AD O‐tau in 3xTg‐AD mice. Intravenous treatment with 77G7 could not effectively inhibit AD O‐tau induced tau pathology in hippocampus of Tg/hTau mouse, but ameliorated hyperphosphorylation of tau induced by AD O‐tau. It has been well established that 0.1–1% of circulating antibodies can enter the brain.^69^, ^70^ A caveat of this scenario is that the therapeutic beneficial effect of tau might vary depending on the availability of the antibody in the brain to inhibit the seeding activity of pathological tau.

In AD and other tauopathies, tau pathology, without exception, is made up of the hyperphosphorylated tau.^71^ Reduction of hyperphosphorylation as well as tau level were shown to inhibit tau pathology and neuronal degeneration and rescue cognitive impairment in transgenic models of mice and rats.^26^, ^48^ Tau pathology can be induced by injection of proteopathic tau aggregates into mouse brain.^25^, ^39^, ^72^ We recently found that injection of AD O‐tau into Tg/hTau mouse hippocampus not only induced tau pathology detected by AT8 immunostaining but also led to site‐specific hyperphosphorylation of tau.^40^ In the present study, intravenous administration of 77G7 decreased or showed a trend to decrease tau phosphorylation. Thus, collectively the present study shows the therapeutic potential of 77G7 in inhibiting tau pathology.

Different from Tg/hTau mice, no increased tau phosphorylation was induced by hippocampal injection of O‐tau into 3xTg‐AD mice, which might be due to the low dosage of AD O‐tau (0.25 μg) and less time (2 months) after AD O‐tau injection. These results suggest that tau aggregation may appear prior to hyperphosphorylation, which was supported by double immunostaining of T22 and AT8 that only large aggregates showed both T22 and AT8 staining, while the small aggregates stained with T22 but not with AT8.

## 5 CONCLUSION

The present study strongly suggests that antibody 77G7 toward the microtubule‐binding repeats of tau has many‐fold higher affinity toward aggregated pathological tau than non‐aggregated normal tau and that 77G7 inhibits the seeding activity in capturing and templating tau aggregation in vitro and in vivo in tau transgenic mice. 77G7 is a promising immunotherapeutic agent to target tau propagation.

## AUTHOR CONTRIBUTION

LL and JM performed the main studies and analyzed the data. DC, NJ, and YCT carried out the study. LL wrote the first draft of the manuscript. CLD, WH, CXG, and KI contributed reagents and animals. CXG, DC, and KI edited the manuscript. FL designed the study, analyzed the data, and wrote the manuscript. All authors read and approved the final manuscript.

## FUNDING INFORMATION

This work was supported in part by New York State Office for People Developmental Disabilities, Nantong University, the Neural Regeneration Co‐innovation Center of Jiangsu Province and by grants from U.S. Alzheimer's Association (DSAD‐15‐363172), Postgraduate Research and Practice Innovation Program of Jiangsu Province (KYCX18_2413) and the National Natural Science Foundation of China (Grants 81872853).

## CONFLICT OF INTEREST

The authors declare that they have no competing interests.

## Data Availability

The datasets generated during and/or analyzed during the present study are available from the corresponding author on reasonable request.
